# Reference Intervals of Factor H and Factor H-Related Proteins in Healthy Children

**DOI:** 10.3389/fimmu.2018.01727

**Published:** 2018-08-02

**Authors:** Anna E. van Beek, Angela Kamp, Simone Kruithof, Ed J. Nieuwenhuys, Diana Wouters, Ilse Jongerius, Theo Rispens, Taco W. Kuijpers, Kyra A. Gelderman

**Affiliations:** ^1^Department of Immunopathology, Sanquin Research and Landsteiner Laboratory of the Academic Medical Centre, University of Amsterdam, Amsterdam, Netherlands; ^2^Department of Pediatric Hematology, Immunology and Infectious Diseases, Emma Children’s Hospital, Academic Medical Centre, Amsterdam, Netherlands; ^3^Sanquin Diagnostic Services, Amsterdam, Netherlands; ^4^Department of Blood Cell Research, Sanquin Research and Landsteiner Laboratory of the Academic Medical Centre, University of Amsterdam, Amsterdam, Netherlands

**Keywords:** normal ranges, complement, complement factor H, factor H-related proteins, pediatrics, diagnostics, reference intervals

## Abstract

Complement is activated as part of the innate immune defense against invading pathogens. Also, it helps to remove apoptotic debris and immune complexes from the circulation. Impaired complement function due to aberrant plasma levels of complement proteins may be indicative for complement-mediated diseases or can be involved in susceptibility for infections. To determine whether plasma levels are abnormal, reference intervals (RIs) are used from adult healthy donors. Since many complement-mediated diseases have an onset during childhood, it is important to know whether these RIs can be extrapolated to children. RIs of Factor H (FH), the crucial fluid-phase regulator, and the FH-related proteins (FHRs), its homologous counterparts, are unknown in healthy children. While FH is measured to diagnose and monitor therapy of patients with atypical hemolytic uremic syndrome, recent studies also implicated increased plasma levels of FHRs in disease. Here, we investigated the levels of FH and FHRs in healthy children using recently developed specific ELISAs. We found that levels of FH, FHR-2, and FHR-3 were equal to those found in healthy adults. Levels of FHR-4A and FHR-5 were lower in children than in adults. However, only the FHR-5 levels associated with age. The RIs of these FH family proteins now serve to support the interpretation of plasma levels in prospective and retrospective studies that can be used for routine diagnostic and monitoring purposes including pediatric patient samples.

## Introduction

Complement is part of innate immunity, comprising a powerful cascade of proteins able to eradicate invading pathogens and is important for removal of apoptotic debris and immune complexes from the circulation. Complement activation is tightly controlled and regulator proteins make sure that bystander damage to healthy host cells is kept to a minimum. Within the population, there is variation in the expression levels of these proteins and other complement components, leading to different steady-state complement activities in healthy individuals (1). Assessment of abnormal circulating levels can help to diagnose complement-mediated diseases such as atypical hemolytic uremic syndrome (aHUS) and differences in expression levels can help in understanding the susceptibility for infectious diseases as described in retrospective studies (2).

To discriminate between normal and abnormal levels, and to interpret retrospective studies, clinical laboratory reference intervals (RIs) are needed. As many complement-mediated diseases can have their onset during childhood, it is important to know whether adult levels can be extrapolated toward pediatric patients. For proteins such as C3 and C4, it has been determined that the normal ranges can be different in childhood compared to adults and between different ethnicities, and as such, adjusted RIs may be used (3–6). No pediatric RIs are known of Factor H (FH) and the FH-related proteins (FHRs), of which their plasma levels associate with various diseases.

Factor H is a crucial regulator of the alternative complement pathway and protects human host cells from unwanted complement activation. Genetic variants in complement regulator FH are associated with multiple diseases. Such variants can either alter protein functionality or induce variation in levels of expression. Many have been described to associate with aHUS or age-related macular degeneration, affecting the regulating function of FH (7, 8). However, some genetic variants result in lower (insufficient) circulating levels of FH (9–11). Differences in steady-state FH protein levels are associated with susceptibility for meningococcal disease and have recently been implicated as a marker of cardiovascular risk in chronic Chagas disease (12, 13). In general, low expression of complement regulators, such as FH, would make an individual more prone for chronic inflammation but more protected against infectious diseases, while high expression rather associates with risk of infectious diseases but less chronic inflammation (14).

Apart from FH, the FH protein family also includes the short splice variant of FH, FH-like-1 (FHL-1), and the FH-related (FHR) proteins, named FHR-1, FHR-2, FHR-3, FHR-4, and FHR-5, all of which are encoded by their own gene. FHR-4A and FHR-4B are the two splice variants of *CFHR4*, but FHR-4A is the only circulating variant found in human serum (15). While FHRs share homology with FH in its surface binding domains, they lack domains similar to SCR1-4 in FH and FHL-1, and for that reason are believed to have no complement-regulatory activity (16). Although limited data are available on the *in vivo* function of FHRs, many have shown associations of complement-mediated diseases with these FHR genes due to their copy number variations (17, 18), internal duplications (19–21), fusion proteins (22–26), or polymorphisms (27–30).

In addition, recent developments in the determination of circulating FHR levels in adults have led to the discovery of new associations with disease. FHR-1 levels were shown to be increased during IgA nephropathy (31, 32), although the authors report much higher levels than we and others have published (33, 34). FHR-3 levels were shown to be elevated during sepsis (35) and in systemic lupus erythematosus, rheumatoid arthritis, and polymyalgia rheumatica (36). Although Schäfer et al. did not find increased FHR-3 levels in aHUS patients, a recent study demonstrated increased levels in a larger, well-characterized cohort (36, 37). FHR-2 and FHR-4A levels have, so far, not been studied except in healthy donors, although FHR-2 and FHR-4A are implicated in the acute phase of bacterial infections (van Beek et al., manuscript in preparation) (15, 33, 38). FHR-5 levels were shown to be decreased in patients with C3 glomerulonephropathy (C3G) (39) and was recently identified as an independent risk factor for IgA nephropathy (32, 40). In summary, assessment of FHR protein levels contributes to the understanding of various diseases.

To investigate whether different RIs should be used for FH and the FHRs in children, we assessed the circulating levels in a cohort of healthy Dutch children and adolescents (all referred to as children), covering various age categories. These RIs now serve to support the interpretation of plasma levels in retrospective studies that include children. Moreover, they can be used for routine diagnostic and monitoring purposes in pediatric patient samples.

## Materials and Methods

### Samples

Serum samples were obtained from anonymous, healthy children from a previous study, in accordance with Dutch regulations and approved by the Sanquin Ethical Advisory Board in accordance with the Declaration of Helsinki (41). Samples from adult healthy donors (*n* = 124 for FH and FHR-3, *n* = 120 for FHR-1, 2, 4A, and 5) were collected and measured during previous studies (15, 33, 35).

### ELISAs

All ELISAs were performed as previously described for an adult healthy donor cohort (15, 33, 35). Briefly, the FH ELISA uses anti-FH.16, a monospecific mAb directed against SCR16-17, as a coat and goat anti-human-FH antiserum as detection. FHR-1/1 homodimers were measured using anti-FH.02 (directed against SCR20 of FH and cross-reactive to SCR5 of FHR-1) both as catching and detecting mAb. FHR-1/2 homodimers were also caught by anti-FH.02, but detected with a commercially available anti-FHR-2 (R&D Systems). FHR-2/2 homodimer levels, as well as total levels of FHR-1 and FHR-2 were calculated based on the observed levels of FHR-1/1 and FHR-1/2 dimers. The FHR-3 ELISA uses anti-FHR-3.1 (cross-reactive to FHR-4A) as a coating mAb and anti-FHR-3.4 (cross-reactive to FH) as a detecting mAb. FHR-4A was measured by catching with the monospecific mAb anti-FHR-4A.04 and detecting with rabbit anti-FHR-3 antiserum. FHR-5 homodimers were measured using two monospecific mAbs, anti-FHR-5.1 and anti-FHR-5.4. Two control sera were included in each plate to ensure limited inter assay variation.

### Statistics

GraphPad Prism software v7 was used to analyze data and perform statistics (GraphPad Software, La Jolla, CA, USA). Significant differences were assessed by unpaired *t*-test. Correlations were assessed with a parametric Pearson’s correlation test.

## Results

With this study, we obtained more insight in the normal ranges of FH and the FHRs in children. For this, we used a cohort of 110 healthy children, of which 53% were females (Table 1). The subjects were evenly distributed across the age categories, aged 7 months up to 251 months (20.9 years) (41). We compared the levels in children to the levels that we previously found in adult healthy Dutch donors (15, 33, 35).

**Table 1:** General cohort characteristics.

Cohort	*n*	Mean age (years)
Total children	110	10.3
Males	52 (47%)	9.2
Females	58 (53%)	11.2

**Age group (years)**	**Males (*n*)**	**Females (*n*)**

0–3	8	1
3–6	13	13
6–9	8	8
9–12	3	9
12–15	8	7
15–18	5	17
18–21	7	3

**Figure 1:**
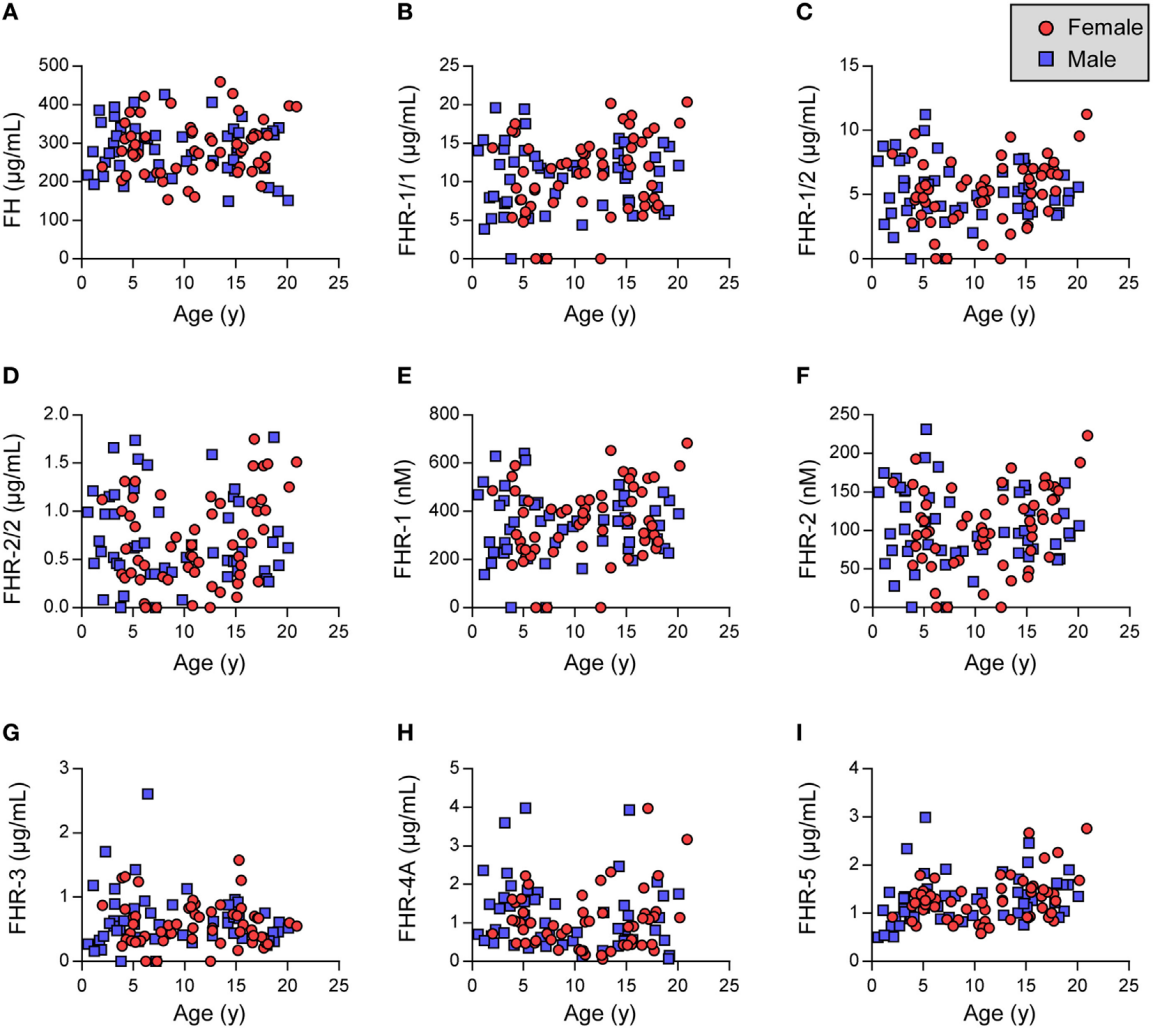
Factor H (FH) family proteins in healthy children. **(A–C,G–I)** Show FH and FHRs as assessed by in-house ELISA. **(D–F)** Indicate calculated FHR-1 and FHR-2 levels. **(D,F)** Samples lacking FHR-1 (likely *CFHR1* deficient) were excluded. Females are indicated by red circles, males by blue squares. Data were analyzed using Pearson’s correlation and unpaired *t*-tests (Table 2).

We investigated the plasma levels of FH and the FHRs in these healthy children (Figure 1). We observed that the levels of FH and FHR-3 were similar between the two genders and independent of age (Figures 1A,G). Indeed, the levels are equal to those previously found in adult healthy Dutch donors (Table 2) (35).

**Table 2:** Factor H (FH) family normal ranges characteristics.

		FH (μg/mL)	FHR-1/1 (μg/mL)^b^	FHR-1/2 (μg/mL)^b^	FHR-2/2 (μg/mL)^a^^,^^b^	FHR-1 (nM)^a^^,^^b^	FHR-2 (nM)^a^^,^^b^	FHR-3 (μg/mL)^b^	FHR-4A (μg/mL)	FHR-5 (μg/mL)
Median	Male	286	11.2	5.1	0.6	351	96	0.58	0.92	1.2
	Female	279	11.5	5.4	0.6	362	105	0.54	0.91	1.2

IQR (25%)	Male	237.5	7.0	3.7	0.39	243	73	0.36	0.54	0.93
	Female	237.3	7.2	3.6	0.34	245	65	0.34	0.46	0.92

IQR (75%)	Male	426.5	13.5	6.7	1.1	439	150	0.81	1.6	1.48
	Female	459.5	14.4	7.0	1.1	469	223	0.74	1.3	1.47

95% range	Male	150–420	0–20	0–11	0–1.8	0–637	0–219	0–2.3	0.1–4.0	0.5–2.8
	Female	157–445	0–20	0–11	0–1.6	0–669	0–209	0–1.5	0.1–3.6	0.6–2.7

Gender difference	Unpaired *t*-test (*P* value)	0.84	0.28	0.75	0.87	0.51	1.00	0.42	0.26	0.99

Children vs age	Pearson *r*	0.00	0.13	0.07	0.03	0.12	0.06	−0.14	−0.03	0.29
	*R* squared	0.00	0.02	0.00	0.00	0.01	0.00	0.02	0.00	0.09
	*P* value	0.97	0.20	0.48	0.73	0.22	0.54	0.15	0.76	0.002

Children vs adults	Unpaired *t*-test (*P* value)	0.655	0.030	0.444	0.870	0.0498	0.873	0.100	<0.0001	<0.0001
	Difference between means (μg/mL)	4.114	1.219	0.2173	−0.0095	32.66 nM	0.9862 nM	0.0952	1.438	0.3863

*^a^Values of FHR-2/2 homodimers, and total levels of FHR-1 and FHR-2 monomers are calculated based on measured levels of FHR-1/1 homodimers and FHR-1/2 heterodimers*.

^b^Donors lacking FHR-1, FHR-2 (in the adult donor cohort) or FHR-3 were excluded from correlations and unpaired t-tests

Next, FHR-1 and FHR-2 were assessed using dimer-specific ELISAs (Figures 1B,C). The levels of FHR-1/1 homodimers were independent of age and gender, although we did find a minor, but significant, difference when comparing the FHR-1/1 levels to adults (Table 2, difference between means = 1.2 µg/mL) (33). FHR-1/2 heterodimers and FHR-2/2 homodimers were also found to be independent of age and gender but were similar to the adult healthy donors (Figure 1D; Table 2). This implied that only the FHR-1 plasma levels differed from the adults. Indeed, when we calculated the concentrations of total FHR-1 and FHR-2 monomers, only FHR-1 levels were significantly lower [difference between means = 1.3 µg/mL (33 nM)] than the healthy adults (Figures 1E,F; Table 2).

We recently demonstrated that FHR-4A is the only circulating form of FHR-4 and that no FHR-4B could be observed in serum (15). Therefore, we measured only FHR-4A in the children and found that FHR-4A levels were lower than expected based on levels found in adult healthy donors (Figure 1H; Table 2). It would, therefore, be expected that the levels showed an association with age. Surprisingly, the FHR-4A levels did neither show an association with age, nor with gender, in the children.

Last, we assessed the levels of FHR-5/5 homodimers. Similar to the other FHR proteins, FHR-5 levels were independent of gender. However, the levels did increase with age (Figure 1I, Table 2), being approximately 0.5 µg/mL lower in the youngest children than in the oldest children. While the younger children indeed showed significantly lower levels, the older children presented with levels equal to the adult healthy donors.

As the *CFHR* genes originated as part of segmental duplications of the *CFH* gene, it would be possible that protein expression is similarly regulated (42). Therefore, we investigated whether FH plasma levels associated with plasma levels of the FHRs. We saw an association between FH and FHR-1/1 homodimer levels in adult donors, when they carry two copies of *CFHR1* (*r* = 0.62, *P* < 0.0001), in contrast to those who carry only 1 copy of *CFHR1* (*r* = 0.09, *P* = 0.67) (33, 35). Children who most likely carry two copies of *CFHR1* [expressing > 10.1 µg/mL FHR-1/1 homodimers, as determined by ROC analysis (area under the curve = 0.97)] showed a similar association (*r* = 0.49, *P* < 0.0001) (33). No association between FH and other FHR levels was noted. As a general conclusion, we observed no remarkable differences compared to adult circulating levels of FH family proteins.

## Discussion

We have determined RIs for FH and FHR-1 to 5 in Dutch healthy children. We were able to interpret the circulating levels of these FH family proteins in relation to adult healthy donors, which we have previously assessed (15, 33, 35). We found differences in some but not all of these proteins in the healthy children when compared with adults.

In contrast to FHR-1, FHR-4A, and FHR-5, no remarkable observations were made when analyzing the circulating levels of FH, FHR-2, and FHR-3. The three proteins were independent of age and gender, confirming a previous study on FH in Brazilian children (43). FH levels were previously found to be low in neonates, suggesting that plasma levels reach adult ranges within the first 6 months after birth (44, 45). Unfortunately, no sera were available from children below the age of 6 months. Future studies should test cord blood and plasma of neonates for the presence of FHRs at birth and early infancy to investigate these protein levels in more detail.

FHR-1 levels were independent of age and gender. We did observe lower FHR-1 levels than previously seen in adults, although the biological relevance may be disputed. FHR-3 levels were also trending toward significance, indicating that a minor difference in the copy number variation in *CFHR3/CFHR1* between the two cohorts might be affecting the results (33, 35).

We found lower FHR-4A levels in children than in adults, even though FHR-4A did not associate with age of the children. Our group demonstrated previously that FHR-4A is stable up to at least 10 freeze-thaw cycles (15). However, we cannot exclude the possibility that long-term storage of these samples may have suffered from breakdown of FHR-4A when kept at −30°C (15). New studies on more recent samples are needed to confirm or disprove this possible explanation.

For FHR-5, we observed an increase with age, indicating that normal ranges for FHR-5 are low in the youngest children and that RIs may need to be adjusted accordingly. As FHR-5 levels positively associated with severity of IgA nephropathy in adults (32), and as IgA nephropathy is the main nephropathy in children (46), measurements of FHR-5 in a pediatric cohort will be highly informative to further study the role of FHR-5 in this nephropathy.

This study represents the most complete assessment of FH family proteins to date in a cohort of healthy children providing RIs. These RIs can now be used to interpret serum levels in prospective and retrospective studies that include children and used for routine diagnostic and monitoring purposes in pediatric patient samples. Ideally, each laboratory should adapt these RIs for their own assays.

## Ethics Statement

Serum samples were obtained from anonymous, healthy children from a previous study, in accordance with Dutch regulations and approved by the Sanquin Ethical Advisory Board in accordance with the Declaration of Helsinki.

## Author Contributions

AB, DW, TK, and KG designed research. AB, AK, SK, and EN performed research. AB, IJ, TR, TK, and KG analyzed data and wrote the paper. All authors critically reviewed the manuscript, gave final approval of the version to be published, and agreed to be accountable for all aspects of the work in ensuring that questions related to the accuracy or integrity of any part of the work are appropriately investigated and resolved.

## Conflict of Interest Statement

The authors declare that the research was conducted in the absence of any commercial or financial relationships that could be construed as a potential conflict of interest.
